# Increased biomass and lipid production of *Ettlia* sp. YC001 by optimized C and N sources in heterotrophic culture

**DOI:** 10.1038/s41598-019-43366-5

**Published:** 2019-05-02

**Authors:** Minsik Kim, Bongsoo Lee, Hee Su Kim, Kibok Nam, Myounghoon Moon, Hee-Mock Oh, Yong Keun Chang

**Affiliations:** 1 Department of Chemical and Biomolecular Engineering, Korea Advanced Institute of Science and Technology (KAIST), 291 Daehak-ro, Yuseong-gu, Daejeon, 34141 Republic of Korea; 2 Department of Microbial and Nano Materials, Mokwon University, 88 Doanbuk-ro, Yuseong-gu, Daejeon, 35349 Republic of Korea; 3 LG Chem, 30 Magokjungang 10-ro, Gangseo-gu, Seoul, 07796 Republic of Korea; 4 Gwangju Bio/Energy R&D Center, Korea Institute of Energy Research (KIER), 270-25 Samso-ro, Buk-gu, Gwangju, 61003 Republic of Korea; 5 Korea Research Institute of Bioscience and Biotechnology (KRIBB), 125 Gwahak-ro, Yuseong-gu, Daejeon, 34141 Republic of Korea; 6 Advanced Biomass R&D Center (ABC), 291 Daehak-ro, Yuseong-gu, Daejeon, 34141 Republic of Korea

**Keywords:** Biofuels, Chemical engineering, Environmental biotechnology, Microbiology techniques, Industrial microbiology

## Abstract

The culture conditions and media composition for the heterotrophic culture of an axenic strain of *Ettlia* sp. YC001 were firstly optimized using the Plackett-Burman design (PBD) and response surface methodology (RSM). The strain successfully showed higher productivity in the basal media without any light illumination at 32.2 to 33.3 °C. The PBD results showed that the most effective components for biomass productivity of *Ettlia* sp. were fructose and yeast extract for sources of C and N, respectively. The RSM results showed an optimal level of 72.2 g/L for fructose and 21.5 g/L for yeast extract, resulting in 46.1 g/L biomass with a lipid content of 13.8% over a course of 9 days. Using a 5 L scaled-up fermentation system for 6 days, the production of biomass and lipids was 7.21 g/L/day and 1.18 g/L/day, respectively. Consequently, heterotrophic cultivation of *Ettlia* sp. YC001 provided much higher production of biomass and lipids than those of autotrophic cultivation. As further research, the use of substitute substrates instead of fructose and yeast extract should be developed to reduce production costs.

## Introduction

Since industrialization in the eighteenth century, the human race has faced drastic climate changes never before seen because of high CO_2_ levels in the atmosphere. In addition, both energy demand levels and human populations are growing exponentially. To address these issues, efforts to develop renewable energy sources have been made in many research areas^1^. However, as a possible solution to climate change, microalgae have attracted recent attention given their prospective productivity per land area and the high quality of the resulting lipids; however, microalgal biomass faces a bottleneck with regard to commercialization^2^. During the microalgal oil (MAO) production process, the cultivation and harvesting steps, in particular, require large-scale cultivation and robust harvesting systems gave the challenge of reducing the cost relative to the area of land used^3^, which is strongly correlated with the productivity of the entire process.

To make the entire process feasible, firstly, applying *Ettlia* sp. YC001 may be a viable solution to the commercialization of microalgal biomass. *Ettlia* sp. is increasingly utilized in the biomass research field given its unusual behavior known as auto-flocculation, which may play a role in the reduction of the cost of the harvesting process. Auto-flocculation refers to a zero-cost harvesting method of species having a floc in various conditions, resulting in sedimentation in a shorter amount of time^4^. The species used have the advantage of auto-flocculation, as well as high lipid content, high CO_2_ tolerance levels, and high concentrations of high-value-added products, such as carotenoids and lutein^5^. Moreover, the extract of *Ettlia* sp. has the potential for use in products intended to shield human skin from UVB^6^.

Secondly, heterotrophic cultivation may be a suitable solution for revising productivity. Heterotrophic growth with a fermenter has several advantages compared to that in a photoautotrophic photobioreactor (PBR). Heterotrophic cultivation allows practically any fermenter to be used, leading to significant capital cost reductions for most processes^7^, whereas mixotrophic and photo-autotrophic cultivation processes may require specific devices at the industrialized scale. In addition, the final concentration of heterotrophic cultivation is usually much higher than that of other cultivation strategies with lower cellular growth rates. As a result, the production rate of biomass, lipids, and co-products is competitive, and the harvesting cost is reduced. Recent research has demonstrated the environmental impact of microalgal cultures by means of life-cycle assessments (LCAs), comparing the environmental effects of heterotrophic fermenters, open raceway ponds, and tubular bioreactors, and concluding that the most eco-friendly cultivation process for biomass is heterotrophic cultivation among the three microalgal cultivation modes^8^. However, a precise LCA of heterotrophic algae still remains constrained by a lack of data^9^; therefore, further research related to the heterotrophic culture of microalgal species should be produced.

However, there are relatively few publications focusing on the heterotrophic culture of *Ettlia* sp., especially considering the only recent separation of *Ettlia* sp. from *Neochloris* sp. according to its uninucleate morphology according to Komarek just 20 years ago^10^. The development of heterotrophic growth and the batch-fed process of *Neochloris oleoabundans* was realized by Morales *et al*.^11,12^, but there were no previous studies on the heterotrophic growth of *Ettlia* sp. Cost-effective cultivation, including not only autotrophic culture but also heterotrophic culture, should be considered. Combining heterotrophic cultivation, which offers high biomass concentrations, with *Ettlia* sp. YC001, a species with advantages of high lipid content, pharmaceutically high-value-added products, including lutein and its own extract, and auto-flocculation (a type of zero-cost harvesting) could drastically lower the cost of the biomass production process. To accomplish this, the heterotrophic cultivation of *Ettlia* sp. should take place under the best possible conditions for growth regardless of the cost efficiency of the cultivation process for the first step, and its performance should be carefully quantified.

Hence, in this research, optimization of the heterotrophic cultivation of *Ettlia* sp. was accomplished to improve biomass productivity. After assessing the possibility, media under the optimized conditions were formulated. First, the possibility of heterotrophic growth was demonstrated with diverse media and temperatures. Second, a media component screening experiment was conducted using the Plackett-Burman design (PBD). Third, the optimization of *Ettlia* sp. with the central composite design (CCD) using the Box-Wilson response surface methodology (RSM), a powerful optimization method, was performed to obtain the optimized media for the species. Finally, biomass and lipid productivity rates with the optimized conditions at flask-scale and 5 L fermenter-scale tests were conducted.

## Results and Discussion

### Identification of the optimal temperature and nutrients for *Ettlia* sp

**Figure 1:**
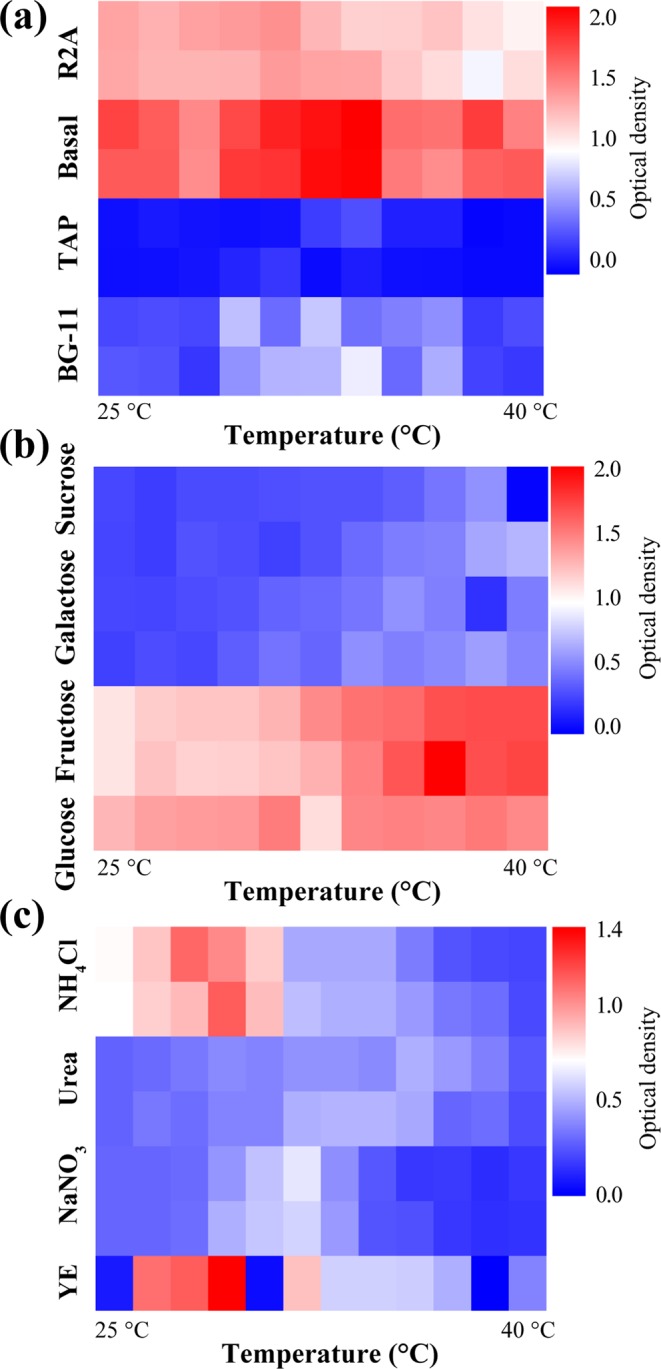
Heat-map of growth in optical density under various nutrient conditions with temperature range from 25 °C to 40 °C. The heterotrophic growth of *Ettlia* sp. in four types of media (**a**), carbon source screening in a selected media (**b**), and nitrogen source test in the same media (**c**).

*Ettlia* sp. was cultivated in 96-well plates with a scale of 300 μl in each well under heterotrophic conditions to screen media for the most suitable method. To adjust the C/N ratio of the four testing media, the elemental analysis of R2A media was conducted and the C/N ratio of the R2A media was 7.98, having 4.55% nitrogen and 36.38% carbon (Table S3). The C/N ratio of the other screening media was adjusted to equalize it, making the growth rates only dependent on nutrient preferences for carbon and nitrogen sources. The cultivation results showed that the basal media had the highest performance at all temperatures, especially in the wells at the 7^th^ column resulting in an OD_682 nm_ of 2.19. The temperature of the 7^th^ well was 32.2 to 33.3 °C (Fig. 1a) on the left side and right side of the wells, respectively.

After choosing the basal media as a base media, the carbon and nitrogen sources were screened for 3 days; fructose, galactose, and sucrose were used to replace glucose at the same concentration of carbon content (Fig. 1b). For the carbon source, fructose and glucose promoted algal growth of more than 1.3 of OD_682 nm_ during the same time, while the maximum growth in sucrose and galactose-containing media was 0.71 of OD_682 nm._ The highest OD_682 nm_ in the plate was observed in fructose, 9^th^ well, with an OD_682 nm_ of 2.06; and maximum OD_682 nm_ in glucose was 1.56. The temperature of the well remained from 32 to 35 °C. It is unique that fructose promoted growth more than glucose because other species show the opposite response, especially considering *Neochloris* sp., which preferred glucose and consumption of fructose was null over a period of 5 days in the work of Morales-Sanchez *et al*.^12^.

For the nitrogen sources, ammonium chloride, urea, and sodium nitrate were tested (Fig. 1c). Regarding the nitrogen source, the NH_4_Cl and yeast extract exceeded 1.0 in OD for 72 h (1.14 and 1.40, respectively). Previous studies on photoautotrophic cultivation of *Ettlia* spp. used sodium nitrate^13^; however, this result showed that yeast extract or NH_4_Cl were more suitable N sources for heterotrophic cultivation of *Ettlia* sp. Yeast extract is a widely used N source in heterotrophic cultivation of other microalgal species^14^. Based on the above-mentioned tests, the best performance was at similar temperatures of 32–34 °C. The maximum specific growth rate between 25 and 35 °C was also observed by da Silva *et al*. for *Desmodesmus subspicatus*^15^, whereas photoautotrophic cultivations of *Ettlia* sp. were usually evaluated at room temperature^13^.

These results provided evidence of the possibility of heterotrophic growth of *Ettlia* sp., narrowing down the optimal C and N media candidates and showing a correlation with the application of an alternative carbon source because of its ability to consume fructose. Consuming fructose is a unique behavior in green microalgae and that could enable the use of alternative carbon sources, such as Jerusalem artichoke (*Helianthus tuberosus*), which can be used as a sustainable biomass feedstock for the biorefinery because of its tolerance for harsher conditions than that of most commercial crops. Furthermore, it has high yields of fresh weight (90 t) per hectare^16^.

### Media component evaluation with the Plackett-Burman design

To confirm the most effective variables in the selected media and to determine the feasibility of alternative carbon and nitrogen sources with the statistical approach, PBD was utilized to assess the biomass concentration after 3 days using a working volume of 100 ml at the flask scale. These results were analyzed using statistical software (MINITAB 14, Minitab Inc., USA).

Table 1 presents the coded simulations and the results in terms of the coded levels, the effects, and the t-test and *P value* outcomes. The R-squared was 0.9994, all factors except for glucose had quantified effects with strong significance at *p* < 0.05; wherever the *P value* of glucose reached 0.1, it may have been caused by the culture conditions of the multiple carbon sources, making it negligible in the results below.

**Table 1:** Analyzed Plackett-Burman design results with R² value of 0.9994.

Variable	Code	Level		Effect	Coefficient	Dry cell weight (g/L)	
		+	−			*T-value*	*P-value*
Intercept				2.4375	0.007	347.13	0.000*
Glucose	G	10	2.5	−0.025	−0.016	−1.78	0.100
Fructose	F	10	2.5	0.161	0.081	11.51	0.000*
Yeast extract	Y	2	0.5	1.692	0.846	120.46	0.000*
NH_4_Cl	N	1	0.25	−0.035	−0.018	−2.49	0.028*
KH_2_PO_4_	K	1.4	0.35	0.075	0.038	5.34	0.000*
K_2_HPO_4_	K2	0.6	0.15	1.145	0.572	81.53	0.000*
MgSO_4_	Mg	0.6	0.15	0.065	0.033	4.63	0.001*
CaCl_2_	Ca	0.05	0.0125	−0.108	−0.054	−7.71	0.000*
FeSO_4_	Fe	0.006	0.0015	0.112	0.056	7.95	0.000*
Trace metals	T	x2	x0.5	0.032	0.016	2.25	0.044*
Vitamin	V	x2	x0.5	−0.038	−0.019	−2.73	0.018*

*Indicates statistically significant at 95% confidence level. Positive (+) and negative (−) levels of each parameter represent the concentration of variables in g/L.

Comparing the effects of the various factors on the biomass outcomes demonstrated that fructose was more suitable as a carbon source rather than glucose, for *Ettlia* sp. given its strong value of effect of 0.162 and the high significance level of 0.000 (*P value*) while glucose had the negative value of effect of −0.025. It is interesting that *Ettlia* sp. YC001 preferred fructose to glucose, whereas most algal species usually prefer the latter. Even *Neochloris* sp. preferred glucose most among C sources^12^, although the species is typically expected to show tendencies similar to those of *Ettlia* sp.^5^. However, Cerón-Garcia *et al*. reported *Phaeodactylum tricornutum* had higher growth with fructose rather than glucose^17^. The consumption of less preferred substrates may have discouraged due to the lack of catalytic enzymes related to uptake and assimilation during the presence of preferred substrate^18^. Or else, the glycolysis pathway of the species may have converted glucose into glucose-6 phosphate (G6P) and after fructose-6 phosphate (F6P), while fructose was directly converted into F6P^19^. Moreover, the lag time of algae might be distinct from each other due to environmental growth conditions. Based on this, revealing the string on sugar consumption mechanism of *Ettlia* sp. YC001 should be done in further research.

On the other hand, the yeast extract was added only in slight amounts at the positive levels, indicating an outstanding value of 1.692 in the PBD assessment. Hence, the yeast extract concentration should have largely increased. Nonetheless, the outstanding positive effect of yeast extract was also observed in the work of Vivek *et al*.^20^. Because these values depended on the coded levels of the factors, fructose did not show a prominent outcome at 0.162 compared to that of the yeast extract, and the concentration of fructose needed to be increased.

Nevertheless, the PBD results indicated that K_2_HPO_4_ had a high effect value of 1.145, the phosphorus content difference would be negligible whereas with a higher concentration of yeast extract in further optimization of media. In addition, the buffering effect in pH from KH_2_PO_4_ and K_2_HPO_4_ also may have a positive role in cellular growth. However, it is clear that the concentration of phosphorus (KH_2_PO_4_ and K_2_HPO_4_) and other nutrient sources with a positive value of effects (MgSO_4_, FeSO_4_, trace elements) should be raised. On the other hand, among the micronutrients in the basal media, CaCl_2_ and vitamin B_1_ demonstrated the negative value of effects of −0.108 and −0.038, respectively. Hence, the optimal media should not include these two elements^21^.

### Screening of lipid extraction methods

The lipid extraction efficiency varies by cell disruption methods^22^. Despite the number of researches has examined the lipid contents of *Etltia* sp.^5,13,23^, the cell disruption method for *Ettlia* sp. has not been assessed comprehensively. To calculate the exact lipid productivity rate in this study, the cell disruption methods of lipid extraction process should be defined before to analyze the content. Based on the Folch method^24^, a few physical treatments were tested and found to be reasonable extraction methods for *Ettlia* sp. These were heating, sonication, a fatty acid methyl ester treatment (acid thermal treatment), methanol soaking, and bead beating. The bead beating was conducted on one sample to fragmentize the dry cell completely until it became completely bleached (5 times). The result of bead beating was considered a positive control.

**Figure 2:**
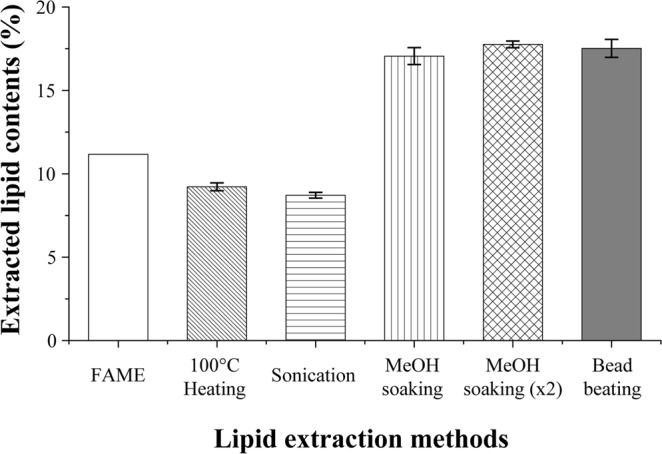
Extracted lipid contents of total lipid extraction methods. The lipid contents are presented as the weight of extracted lipids over the weight of dry biomass. MeOH soaking (x2) implies soaking twice before extraction.

Figure 2 shows the results for the extracted lipids in various methods. The groups, which underwent heating and sonication, showed lower lipid contents of 9.2 ± 0.2% and 8.7 ± 0.2%, respectively, though the FAME contents were higher (11.2%). Here, the FAME content does not include the weight of glycerol, which is a result of transesterification. Its value is usually lower than that of the total for lipids, indicating that approaches based on the Folch method cannot be used to extract lipids out of cells sufficiently. Furthermore, the methanol soaking method led to a high extraction yield (17.1 ± 0.5% of the contents the first time, 17.8 ± 0.2% the second time), reaching that of the positive control, i.e., the bead beating method (17.5 ± 0.5%). Yang *et al*.^25^ demonstrated the extraction effect using only alcohol alone (ethanol), and it may cause a higher extraction efficiency of methanol-soaked experimental groups. However, bead beating showed lower disruption efficiency than sulfuric acid and high-pressure homogenization according to Halim *et al*.^22^. Regardless, the previous study was limited in the bead beating operation time to 4 min. Additionally, considering that the results of this study showed that bead beating had higher extraction efficiency than that of acid-thermal treatment, the methanol-soaking method (extracted twice) was chosen as the most appropriate extraction method for *Ettlia* sp. in this work.

### Optimization of the heterotrophic culture conditions

Whereas the PBD experiment determined the effects of the factors on the biomass, the concentration of each component in the media remained uncharted. As the PBD results were not sufficient to achieve this, RSM experiments were performed. Yeast extract is not a defined type of media (it contains magnesium, phosphorus, and even carbon); therefore, it must be adjusted to an amount with only a carbon source. The amounts of all of the nutrients that had positive effects (K_2_HPO_4_, KH_2_PO_4_, MgSO_4_·7H_2_O, FeSO_4_·7H_2_O, trace metals) were doubled. Center points and intervals of CCD were revised continuously to find the peaks of the response surface.

The final experimental set with 9 days of cultivation is described in Table S4, and the results of an analysis of variance (ANOVA) test is described in Table 2. The F-value and the P value of the RSM model were 21.42 and 0.005, respectively, which indicated that the model was significant with only a 0.05% chance that the F-value was large because of noise. Additionally, the P value of “Lack of fit” was 0.1236, greater than 0.05 and it also showed that the lack of fit was not significant relative to pure error, and there is a 12.36% of chance that the F-value from “Lack of fit” was large because of noise. In addition, adequate precision (12.623) was greater than 4; thus, this model could be used to navigate the design space^20^. The P values of most parameters were below 0.05, indicating that each variable and its coefficient were very significant except for B (yeast extract) and AB.

**Table 2:** ANOVA table for the biomass RSM model.

Source	Sum of squares	Degree of freedom	Mean square	*F-Value*	*P-value*	Remarks
Model	271.15	5	54.23	21.42	0.0005	Significant
A-Fructose	19.00	1	19.00	7.50	0.0337	
B-Yeast extract	5.08	1	5.08	2.00	0.2152	
AB	12.89	1	12.89	5.09	0.0666	
A^2^	227.90	1	227.90	90.03	<0.0001	
B^2^	19.84	1	19.84	7.83	0.0310	
Residual	19.16	7	2.74			
Lack of fit	13.99	3	4.66	3.61	0.1236	Not significant
Pure error	5.17	4	1.29			
Cor. total	290.31	12				

Quadratic equation is y = 46.06 + 1.5409 * F − 5.7238 * F^2^ − 1.6887 * Y^2^ − 1.795 * F * Y and the optimal point was F = 72.2 g/L, YE = 21.5 g/L, with biomass of 46.2 g/L and desirability of 0.892.

R^2^ of reduced model is 0.9165, Adjusted R^2^ = 0.8748, Predicted R^2^ = 0.7320, and adequate precision = 12.623.

Because the P values above 0.10 indicated that the variable was not significant, a quadratic equation model was generated eliminating the only non-significant term, B.

1 $${\rm{y}}=46.06+1.54\ast {\rm{F}}-5.72\ast {{\rm{F}}}^{2}-1.69\ast {{\rm{Y}}}^{2}-1.80\ast {\rm{F}}\ast {\rm{Y}}$$

**Figure 3:**
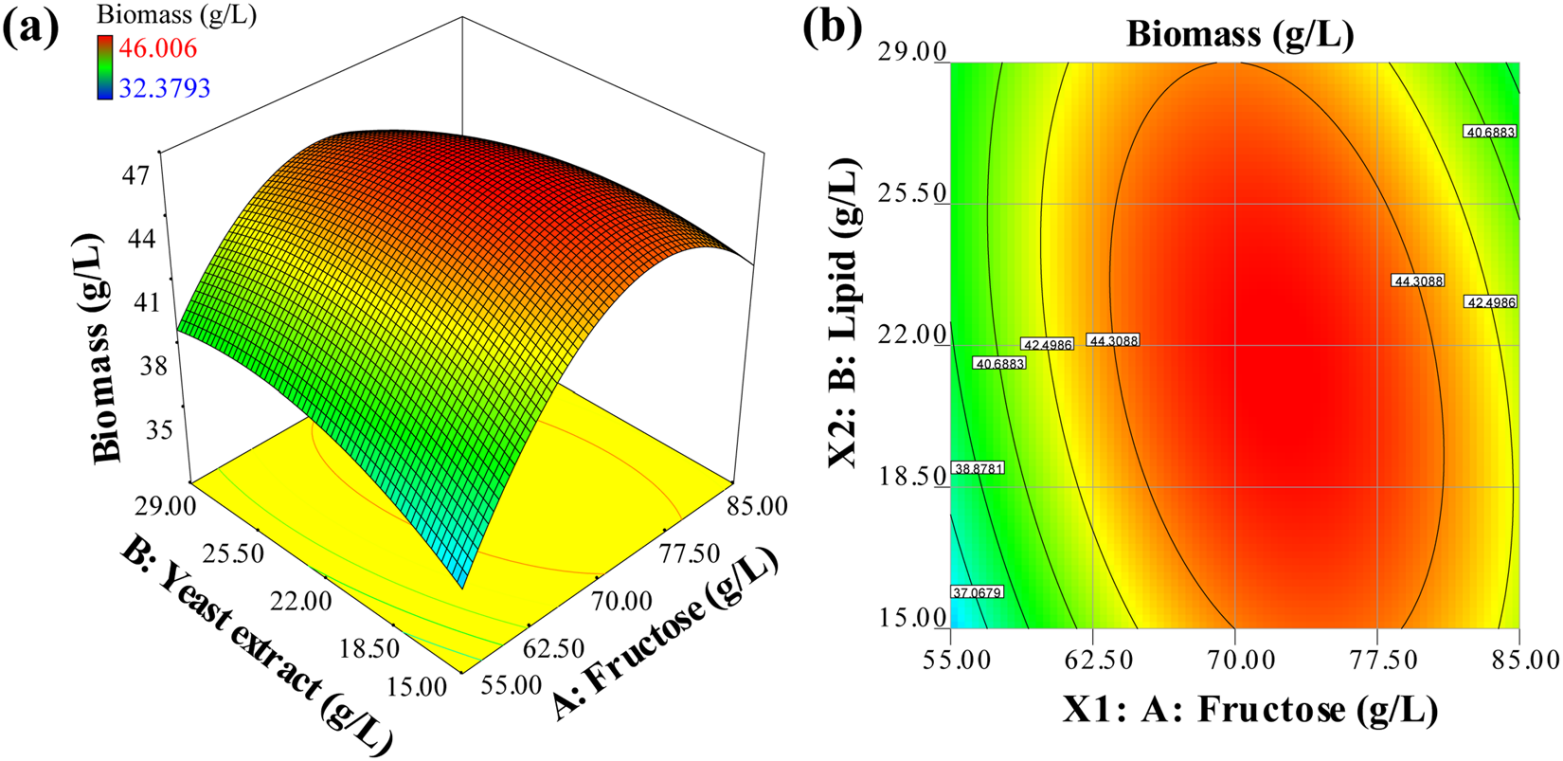
RSM result in a 3D surface (**a**) and a contour (**b**). The biomass response from the interaction of fructose concentration and yeast extract concentration in g/L each. The peak of the surface is the optimal point, which is located at fructose 72.2 g/L, yeast extract 21.5 g/L, with a biomass response of 46.1 g/L.

Here, y is the expected biomass concentration, F denotes fructose, and Y is yeast extract. The actual value of biomass responses and predicted biomass responses using Eq. (1) are represented in Table S4, with the last two columns showing the lipid responses and predicted values of the lipids. The R-squared value of the reduced model (B term eliminated) was 0.9165, which is reasonable; the predicted R-squared of 0.7320 was in reasonable agreement with the adjusted R-squared of 0.8748. Thus, the statistical approach demonstrated the significance of the surface model and it was reasonable to assume the optimum^21^. Using this model, the optimal point of biomass concentration could be derived. Solving the partial differential equation suggested an optimal point with fructose = 72.2 g/L, yeast extract = 21.5 g/L, and biomass of 46.1 g/L. The RSM result of the 3-D surface and contour shown in Fig. 3 clarifies the existence and location of the optimum concentration at the peak of the response surface. This matches the result of Doucha *et al*.^26^, wherein *Chlorella vulgaris* had high-glucose tolerance up to 80 g/L, whereas *Chlorella protothecoides* had an inhibition from 60 g/L of glucose^27^. Hence, the substrate tolerance depends on the algal species, and the results of the RSM fit in a reasonable concentration. Furthermore, the lipid content of all experimental set was analyzed and the highest nitrogen-containing media resulted in 12.9%, while the maximum lipid content among the experimental group was 15.8%. Notwithstanding the contrast of lipid content, fructose and yeast extract concentrations of lipid response were very close to that of the biomass response (Fig. S2, Table S5), being 72.7 g/L for the fructose concentration and 21.3 g/L for the yeast extract concentration; hence, the lipid content difference between culture conditions was negligible under the effect of the immense biomass productivity distinction and the optimal point of biomass productivity is adjacent to that of lipid productivity.

From the results of the PBD and RSM assessments, for both biomass and lipids, optimal media for the heterotrophic cultivation of *Ettlia* sp. was developed. The optimal media is as follows: fructose at 72.2 g/L, yeast extract 21.5 g/L, KH_2_PO_4_ 1.4 g/L, K_2_HPO_4_ 0.6 g/L, MgSO_4_·7H_2_O 0.6 g/L, FeSO_4_·7H_2_O 6.0 mg/L, A5 trace metal solution 2 ml/L (H_3_BO_3_ 5.72 mg/L, MnSO_4_·7H_2_O 5.00 mg/L, ZnSO_4_·7H_2_O 444 μg/L, CuSO_4_·5H2O 158 μg/L, and Na_2_MoO_4_·2H_2_O 42 μg/L).

**Figure 4:**
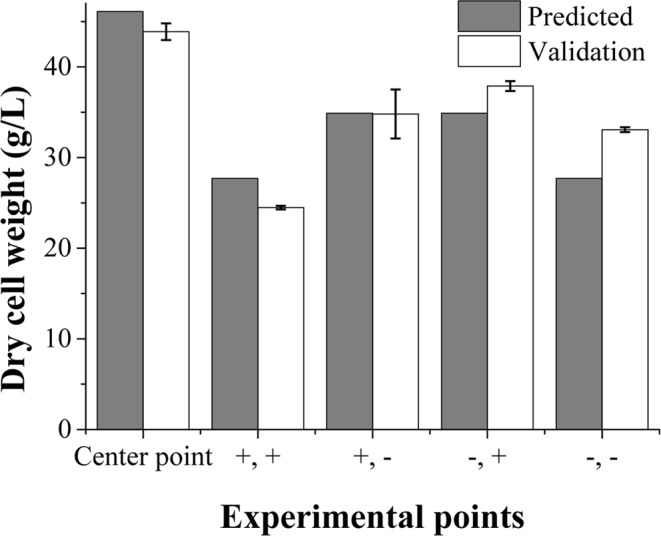
Final biomass concentration of the RSM validation test. Center point indicates the optimal point (72.2 g/L fructose, 21.5 g/L yeast extract). Former (+) sign stands for 93 g/L and (−) sign for 50 g/L fructose, and later (+) sign indicates 31 g/L and (−) sign is 11 g/L yeast extract.

To cross check the optimum levels of this media, a validation test was conducted (Fig. 4) with a new center point and four unchecked spots with the points of an out-cross square of the circle of CCD (Fig. S1). Here, the center point was revised to the optimum point (fructose 72 g/L, and yeast extract 21.5 g/L), and the concentration of validation points were as follows: (+, +), (fructose 93.4 g/L, yeast extract 31.4 g/L); (+, −), (93.4 g/L, 11.6 g/L); (−, +), (51.0 g/L, 31.4 g/L); and (−, −), (51.0 g/L, 11.6 g/L). The test showed a result of 43.88 ± 0.92 g/L for the biomass concentration with a predicted value of 46.2 g/L at the optimal point; thus, the validation and model were significant. However, there were possibilities of errors and exaggerations of the predicted response from the quadratic equation because of water evaporation, which occurred during the working time of 9 days.

### Cultivation in a 5 L fermenter

To confirm the maximum biomass productivity in the optimal media and temperature conditions with an improved oxygen transfer rate of 1 vvm, with positive pH control using NaOH, and the use of condensing evaporated vapors, cultivation with a working volume of 3 L was conducted. The temperature and pH range were controlled automatically at 33 °C and 6.8 to 7.2, respectively, and aeration was controlled and increased to maintain dissolved oxygen until it reached the maximum flow rate of 1.6 vvm. The lights used had hexagonal stainless jackets.

**Figure 5:**
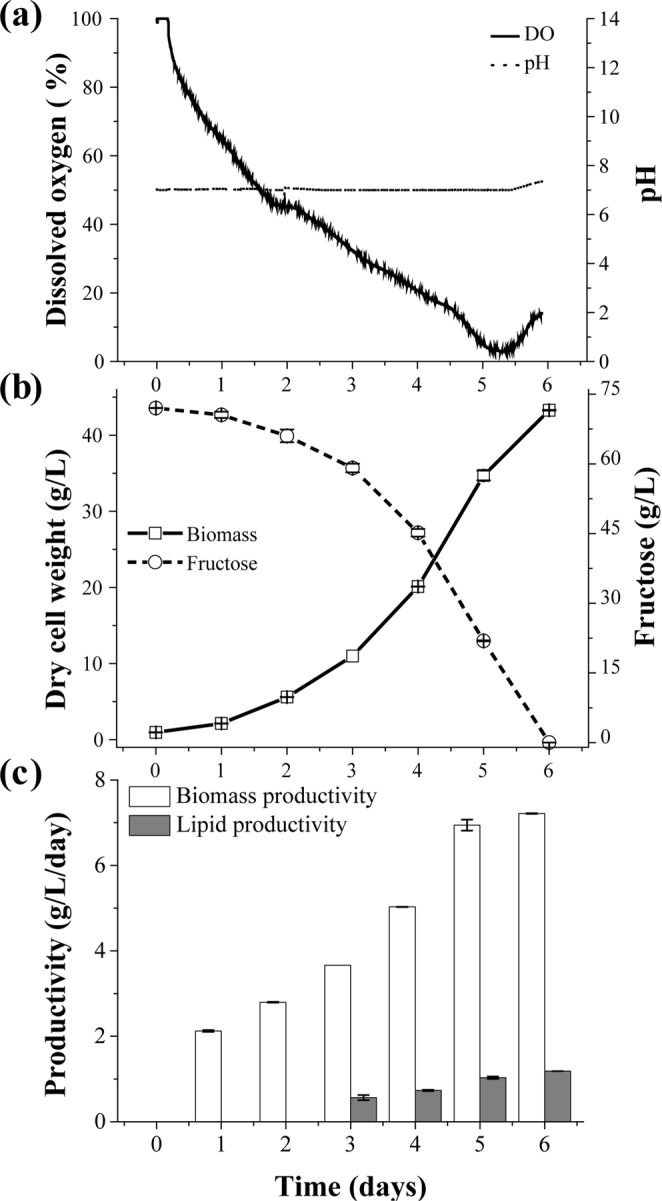
5 L Fermenter experiment for 6 days with optimized media. (**a**) Solid line indicates dissolved oxygen in percentage and dotted line indicates pH. (**b**) Solid line indicates biomass growth in g/L, dotted line indicates fructose concentration in g/L. (**c**) Grey bar indicates lipid productivity per day and white bar is biomass productivity in g/L/day.

The results of 6 days of cultivation are shown in Fig. 5 and the productivity of FAME and lutein from the 5 L fermenter are shown in Table 3. The DCW was calibrated by means of water evaporation, with an additional experiment to measure evaporation per day under fixed conditions (approximately 40 ml/day). During the cultivation, the dissolved oxygen remained before depletion of the carbon source (fructose) and pH was maintained near 7 (Fig. 5a).

**Table 3:** Productivity of lutein and fatty acid methyl ester from 5 L fermenter operation (from day 4 to day 6).

Product from biomass	Productivity (mg/L/day)		
	Day 4	Day 5	Day 6
Lutein	4.60 ± 0.00	6.06 ± 0.80	5.99 ± 0.00
C10:0	98.06 ± 0.05	88.23 ± 1.64	98.70 ± 0.08
C16:0	121.86 ± 0.06	177.31 ± 3.29	202.65 ± 0.17
C16:1	20.04 ± 0.01	28.20 ± 0.52	39.99 ± 0.03
C18:0	18.93 ± 0.01	37.54 ± 0.70	16.14 ± 0.01
C18:1n9c	108.80 ± 0.05	151.25 ± 2.83	32.58 ± 0.03
C18:2n6c	127.87 ± 0.06	159.21 ± 2.95	198.59 ± 0.17
C18:3n3	26.07 ± 0.01	33.15 ± 0.62	175.26 ± 0.15
C22:2	162.05 ± 0.08	223.61 ± 4.15	38.13 ± 0.03

Regarding this as a zero-stress condition, the biomass productivity reached its maximum value of 43.3 g/L on day 6 (Fig. 5b), with the value recorded is 47.8 g/L of biomass without considering evaporation; nearly identical to the optimal biomass productivity in the RSM case. The fructose in the supernatant was also depleted on day 6, such that the batch cultivation of the species ended, resulting in cultivation time cut to two over three, from 9 days to 6 days compared to flask-scale cultivation.

Under all of the conditions above, the daily biomass productivity was 7.21 g/L/d (Fig. 5c) on day 6. Previously published works of *Ettlia* spp. reached 1.48 g/L/day of biomass productivity with photoautotrophic chemostat according to Seo *et al*.^13^, and Kam *et al*.^28^ achieved 1.17 g/L/day with mixotrophic cultivation by brewery wastewater. Furthermore, this greatly exceeding the heterotrophic biomass productivity of 1.9 g/L/day of *Neochloris oleoabundans*^11^. This is a greatly increased result regarding the productivity of *Ettlia* spp., and also a remarkable value in productivity of microalgal heterotrophic batch cultivation.

Meanwhile, lipid contents and profiles were analyzed and recorded at 14.8% for day 5 and 16.4% for day 6. These total lipid contents were less than those in the previous reports of 51% lipids from Yoo *et al*.^29^ and 57% from Lee *et al*.^30^. However, those results were derived under relatively limited nutrient conditions. The biomass productivity of Yoo *et al*. was 0.1 g/L/day^5^ and Lee at al. conducted 30 days of N-starvation condition under the light condition to induce carotenoids and to increase the lipid content^30^. Furthermore, lipid-RSM result (Fig. S2) demonstrated the excessive biomass productivity during exponential phase increased lipid productivity at the same time. Additionally, *Ettlia* spp. studies that have relatively high biomass productivity of over 1.0 g/L/day had under 20% lipid content (19% from Kam *et al*.^28^, 20% from Seo *et al*.^13^). Hence, it appears that there was not enough time to convert the carbohydrates into lipids^31^. To induce lipid content in nitrogen-limited stationary phase, a process development of a fed-batch process or two-step strategy would be necessary, risking the loss in productivity due to longer cultivation time. While photoautotrophic and mixotrophic cultivation utilizes CO_2_ as a carbon source, which can be supplied from ambient air, sugar should be supplied to heterotrophic mode to maintain the cell’s activity and mass during the inducement. Remarking that, lipid productivity reached 1.02 g/L/day on day 5 and 1.18 g/L/day on day 6 (Fig. 5c), which is the highest value of *Ettlia* sp. among the featured results in Table 4. In addition, it maintained the ratio of fatty acids and had a tendency similar to those in previous works^5^.

**Table 4:** Productivity of *Ettlia* sp. cultivation includes lipid content, lipid productivity, and biomass productivity with various cultivation modes.

Species	Cultivation mode	Lipid content (%)	Lipid productivity (g/L/d)	Biomass productivity (g/L/d)	Working volume	Reference
*Ettlia* sp. YC001	Photoautotrophic	31	0.15	0.85	400 mL	Lee *et al*.^30^
	Wastewater mixotrophic	19	0.23	1.17	100 mL	Kam *et al*.^28^
	Photoautotrophic chemostat	20	0.29	1.48	800 mL	Seo *et al*.^13^
	Photoautotrophic chemostat	51	0.05	0.1	5 L	Yoo *et al*.^29^
	Waste water mixotrophic	14	0.09	0.67	50 mL	Moon *et al*.^23^
*Ettlia oleoabundans*	Photoautotrophic	1.5	0.01	0.31	66 mL	Yang *et al*.^46^
*Neochloris oleoabundans* UTEX 1185	Heterotrophic exponential fed-batch	54	1.02	1.9	3 L	Morales *et al*.^11^
*Ettlia* sp. YC001	Batch fermenter	16.4	1.18	7.21	3 L	This work

During the fermenter-scale experiment, the FAME compositions on day 4 and day 5 appeared to be similar, though a rapid change occurred on day 6 with increases in C16:0 (palmitic acid), C18:2n6C (linoleic acid), and C18:3n3 (eicosapentaenoic acid), and decrease in C22:2 (docosadienoic acid) likely because of the limitations of the nutrient (fructose) and pH changes in the early stationary phase. Moreover, the lutein content continuously decreased along cultivation; however, the productivity was 6.06 ± 0.11 g/L/day at day 5, is near the maximum amount reported (6.1 mg/L/day)^30^ because of an excessive amount of biomass productivity. In this work, the productivity of the biomass and lipids each reached record highs relative to all previous references to *Ettlia* spp. cultivation.

In comparison to all microalgal species, *Chlorella protothecoides* was more optimized, showing better productivity of 8.65 g/L/d with a semi-continuous process according to Ceron-Garcia *et al*.^32^. However, it is hard to directly compare semi-continuous culture with batch cultivation. Among batch cultivations of microalga species, on the other hand, this work had the densest concentration of biomass productivity. In batch cultivation, *C*. *protothecoides* showed 3.28 g/L/day of biomass productivity according to Ceron-Garcia *et al*.^32^, 1.12 g/L/day in Gao *et al*.^33^, 3.48 g/L/day in Cheng *et al*.^14^, and 3.5 g/L/day in Mu *et al*.^34^ (Table S6)^35–37^.

Because the optimization was limited in batch fermentation and as the microalgal growth stopped during the exponential phase with the depletion of fructose without a saturation curve for the biomass, it may be possible to achieve higher productivity with the calibration of the optimal point with a batch-fed or continuous process at the fermenter scale. However, the optimal media formulated in this work was very expensive and had high fructose and yeast extract concentrations. To address this, the hydrolysis of Jerusalem artichoke^38^ could be used for fructose production and the hydroxylation of the lipid-extracted biomass could be used to replace the yeast extract^39^. These efforts could be attempted in future work.

## Conclusions

The heterotrophic cultivation of axenic *Ettlia* sp. YC001 was confirmed to obtain higher biomass and lipid production. The most effective components for biomass productivity of *Ettlia* sp. were fructose and yeast extract for C and N sources, respectively, based on the Plackett-Burman design (PBD). In a 5 L fermenter experiment, biomass and lipid productivity reached 7.21 and 1.18 g/L/day, respectively, with a cultivation time of 6 days. As a result, optimizing heterotrophic cultivation of *Ettlia* sp. YC001 provided much higher production of biomass and lipids than did those of autotrophic and mixotrophic cultivation. For further research, utilizing substitute substrates instead of fructose and yeast extract should be developed to reduce the production cost for mass cultivation.

## Materials and Methods

### Seed culture of *Ettlia* sp

The axenic freshwater microalga *Ettlia* sp. YC001, a promising biofuel feedstock, was kindly provided by the Korea Research Institute of Bioscience and Biotechnology (KRIBB). It was used after isolation according to Yoo *et al*.^5^ and established in an axenic culture with a micropipette technique devised by Vu *et al*.^40^. The strain was cultivated in a 1 L filter-cap bottle with 500 ml of TAP media at 200 rpm and 25 ± 1 °C for use as a seed culture and was inoculated at 3% in v/v with 0.1 g/L of inocula biomass for the PhotoBiobox system with PBD and at 1 g/L for the RSM and fermenter experiments. The composition of the TAP media was 2.42 g/L H_2_NC(CH_2_OH)_3_ (Tris base), 375 mg/L NH_4_Cl, 100 mg/L MgSO_4_·7H_2_O, 50 mg/L CaCl_2_·2H_2_O, 288 mg/L K_2_HPO_4_, 144 mg/L KH_2_PO_4_, 50 mg/L Na_2_EDTA·2H_2_O, 22 mg/L ZnSO_4_·7H_2_O, 11.4 mg/L H_3_BO_3_, 5 mg/L MnCl_2_·4H_2_O, 5 mg/L FeSO_4_·7H_2_O, 1.6 mg/L CoCl_2_·6H_2_O, 1.6 mg/L CuSO_4_·5H_2_O, 1.1 mg/L (NH_4_)_6_Mo_7_O_24_·4H_2_O, and 1 ml/L acetic acid^41^.

### Screening culture conditions using PhotoBiobox

PhotoBiobox (Shinwha Science, Republic of Korea) enables screening under multiple conditions in well plates with various light and temperature conditions^42^. To screen base media and manage the cultivation temperature simultaneously, four different types of conventional heterotrophic media (basal media without sea salt, TAP, BG-11 with glucose, and R2A) and temperatures ranging from 25 °C to 40 °C were tested in 96-well plates for three days. The C/N ratio of all media was fixed at that of R2A according to analysis with an elemental analyzer (FLASH 2000 Series CHNS/O analyzer, Thermo Scientific, USA). The standard concentration of BG-11 was NaNO_3_ 1.5 g/L, K_2_HPO_4_ 0.04 g/L, MgSO_4_·7H_2_O 0.075 g/L, CaCl_2_·2H_2_O 0.036 g/L, citric acid 0.01 g/L, ferric ammonium citrate 0.006 g/L, Na_2_·EDTA 0.001 g/L, Na_2_CO_3_ 0.02 g/L, and 1 mL of trace metal solution per liter. The trace metal solution contained H_3_BO_3_ 61.0 mg/L, MnSO_4_·H_2_O 169.0 mg/L, ZnSO_4_·7H_2_O 287 mg/L, CuSO_4_·5H_2_O 2.5 mg/L, and (NH_4_)_6_MoO_4_·4H_2_O 12.5 mg/L^43^. The composition of the basal media per liter was 30 g glucose, 4 g yeast extract, 0.7 g KH_2_PO_4_, 0.3 g K_2_HPO_4_, 0.3 g MgSO_4_·7H_2_O, 25 mg CaCl_2_·H_2_O, 3 mg FeSO_4_·7H_2_O, and 1 ml A5 trace metal solution (286 mg H_3_BO_3_, 250 mg MnSO_4_·7H_2_O, 22.2 mg ZnSO_4_·7H_2_O, 7.9 mg CuSO_4_·5H_2_O, 2.1 mg Na_2_MoO_4_·2H_2_O, and 100 ml distilled water) with NaCl and vitamin B_1_ removed^44^. For the last test, R2A broth mix (Cat. No. of R0005, Teknova, Canada) was used. After selecting the media, four different carbon sources (glucose, fructose, sucrose, and galactose) and four nitrogen sources (yeast extract, NH_4_Cl, urea, and NaNO_3_) were tested again with the aforementioned temperature conditions in the PhotoBiobox system. All media were sterilized with 0.22 μm bottle-top vacuum filters (Corning, USA) to avoid caramelization. The cellular growth was observed by measuring the optical density (OD) at 682 nm using a microplate reader (BioTek, Germany). The last column of all plates remained blank, and the temperature of each well was measured with an infrared thermometer (62 MAX, Fluke, USA)

### Optimizing medium components using the Plackett-Burman design

Quantification of the exact effects of the sources in the basal media on the algal growth was conducted with a PBD experiment. Factors for the design were determined by the PhotoBiobox results, and the total number of experiments was fixed at k + 1, where k represents the number of factors. In this case, k was 11 (nine basal media components, plus fructose and NH_4_Cl). Each factor was tested at two levels: +1 for a high value and −1 for a lower value. The coded experimental set and levels of parameters were determined to refer to Vivek *et al*.^20^. Details of parameters and levels are given in Table 1, and the experimental set is in Table S1. Twenty-four experimental sets were coded in duplicate for all 11 factors, with the biomass response. All experiments were conducted on the scale of the experimental set in 250 ml baffled flasks with a working volume of 100 ml. The flasks were locked with open-filtered caps, placed in a heterotrophic incubator, and then processed while shaking at 125 rpm at the optimum temperature of 33 °C according to the PhotoBiobox results.

### Media optimization using the central composite design based response surface methodology

According to the defined effects from PBD, the two major components that had more significant positive values pertaining to the effects of PBD were selected to optimize the central experimental design and calculations, referring to Bezerra *et al*.^45^ and Fang *et al*.^21^. The center points of the experimental design were set as follows: fructose 70 g/L and yeast extract 22 g/L. Table S2 presents specific levels of all coded factors in terms of g/L. The number of replications in the experiment was set to 1, and five center points and eight non-center points were assessed. For the calculations, the biomass concentrations were used as the responses, with these values fitted by a second-order polynomial equation. The entire process above was analyzed with a statistical program (Design Expert 11.0.0, Stat-Ease, USA), and cultivation following the coding level was done at the flask scale with a working volume of 100 ml. All the experimental points were duplicated and analyzed with average values. Details of the incubation step followed that of the PBD assessment. After the CCD experiment, an analysis of variance (ANOVA) test was conducted with the same program. A validation test was also conducted as in previous optimum conditions with a new center point and the points of an out-cross square of the CCD (interval of 1.414, four points of ++, +−, −+, −) (Fig. S1).

### Cultivation in a 5 L fermenter

A fermenter-scale experiment was conducted with a 5 L fermenter (CNS, Republic of Korea) with a working volume of 3 L. Inoculum was prepared with newly optimized media with a single colony at the flask scale. Filtered media were inserted into the fermenter for an inoculum amount of 1 g/L. The culturing temperatures of the fermenter and condenser were maintained at 33 °C with a water jacket and at 2 °C with a water bath, respectively. The total pH and dissolved oxygen (DO) values were logged in the operating program. An automated pH control system maintained the pH in the range of 6.8 to 7.2 with feedback control using 2N NaOH. Aeration with ambient air was manually increased according to the current DO level to 1.6 vvm (5 L/min). Agitation was conducted at 300 rpm. The dry cell weight (DCW) data for the fermenter experiment was adjusted according to the water evaporation rate.

### Analyses of DCW, fructose concentration, and lutein

The DCWs of all samples were measured after freeze-drying. In each case, 5 ml of the sample was centrifuged at 6,666 × *g* in a Combi R515 (Hanil Scientific Inc., Republic of Korea). Each was also washed with distilled water in the middle of the process and then centrifuged again. The resulting pellets were dried in a HyperCOOL 8080 freeze-drier (Hanil Scientific Inc., Republic of Korea) for 4 days after being kept in a deep freezer at –70 °C for 1 day. The concentration of the carbon source in the supernatant was measured with high-performance liquid chromatography (HPLC) Ultimate 3000 RSLC Nanosystem (Thermo Scientific, USA). The lutein content was analyzed with the same machine, following the methods of Lee *et al*.^30^.

### Lipid extraction and fatty acid methyl ester (FAME) composition

The lipid content was analyzed after screening with the most effective extraction technique based on the Folch method considering the work of Sung *et al*.^24^. Pellets from every experimental group were weighed to assess the DCW, treated with a chloroform-methanol (2:1) solution to extract the lipids, and then sonicated for 2 h for the first experimental group, and heated for 2 h at 100 °C for the second experimental group to provide a harsher condition. For the third and fourth experimental groups, methanol without chloroform was used to soak the dry biomass, with chloroform added later. For positive control, to determine the maximum lipid yield from the biomass, a methanol soaked-bead beating process was utilized. After the above extraction processes, distilled water was added to each group to achieve phase separation, and the lower chloroform was drawn with a syringe, placed on an aluminum dish, and dried overnight. The same procedure was used with the residual biomass to extract whole lipids. The total lipid content was calculated with the following equation:

2 $${\rm{Total}}\,{\rm{lipid}}\,{\rm{content}}\,( \% )=\frac{({W}_{L}-{W}_{D})\times {V}_{C}}{{V}_{P}\times {W}_{S}}\times 100$$

Here, *W*_*L*_ is the weight of the dish with the lipids, *W*_*D*_ is the weight of the empty dish, *W*_*S*_ is the weight of the biomass, *V*_*P*_ is the drawn chloroform phase, and *V*_*C*_ is the total volume of the chloroform added during the extraction process.

The FAME components were analyzed by means of gas chromatography. To do this, 10 mg of pellets were weighted and soaked with a solution of methanol and C17 standard solution (5 mg/1 ml chloroform), after which they were mixed. Subsequently, the mixture was reacted in 15% sulfuric acid at 110 °C for 20 min in a heat block. Finally, distilled water was added, the mixture was centrifuged, and the lower chloroform was drawn with a syringe. This was then filtered and analyzed with gas chromatography (Agilent 7890, USA) and a flame ionization detector (FID) with a HP-INNOWAX column (30 m × 0.32 mm × 0.5 μm).

## Supplementary information

Supplementary information

## Data Availability

The datasets generated during and/or analyzed during the current study are available from the corresponding authors on reasonable request.
