# Supplementary material for: Increased biomass and lipid production of Ettlia sp. YC001 by optimized C and N sources in heterotrophic culture
Source: Sci Rep. 2019 May 2;9:6830. doi: 10.1038/s41598-019-43366-5 (PMC6497641; doi:10.1038/s41598-019-43366-5)

Supplementary information

**Increased biomass and lipid production of *Ettlia* sp. YC001 by optimized C and N sources in heterotrophic culture**

Minsik Kim ^a^, Bongsoo Lee ^b^, Hee Su Kim ^a^, Kibok Nam ^c^, Myounghoon Moon ^d^, Hee-Mock Oh ^e, *^ and Yong Keun Chang ^a, f, *^

^a^ Department of Chemical and Biomolecular Engineering, Korea Advanced Institute of Science and Technology (KAIST), 291 Daehak-ro, Yuseong-gu, Daejeon 34141, Republic of Korea

^b^ Department of Microbial and Nano Materials, Mokwon University, 88 Doanbuk-ro, Yuseong-gu, Daejeon 35349, Republic of Korea

^c^ LG Chem, 30 Magokjungang 10-ro, Gangseo-gu, Seoul 07796, Republic of Korea

^d^ Gwangju Bio/Energy R&D Center, Korea Institute of Energy Research (KIER), 270-25 Samso-ro, Buk-gu, Gwangju 61003, Republic of Korea

^e^ Korea Research Institute of Bioscience and Biotechnology (KRIBB), 125 Gwahak-ro, Yuseong-gu, Daejeon 34141, Republic of Korea

^f^ Advanced Biomass R&D Center (ABC), 291 Daehak-ro, Yuseong-gu, Daejeon 34141, Republic of Korea

* Corresponding author
email: heemock@kribb.re.kr (H.-M. Oh), ychang@kaist.ac.kr (Y.K. Chang)
Fax: +82-42-860-4594 (H.-M. Oh), +82-42-350-3910 (Y.K. Chang)
Tel: +82-42-860-4321 (H.-M. Oh), +82-42-350-3927 (Y.K. Chang)

Table S1. Plackett-Burman design, coded experiments, and the biomass responses. The positive number represents the higher concentrations of the parameter and the biomass is the actual result in g/L.

| Std. order | Run order | Pt. type | Block | Glucose | YE | KH_2_PO_4_ | K_2_HPO_4_ | MgSO_4_ | CaCl_2_ | FeSO_4_ | Fructose | NH4Cl | TM | Vitamin | Biomass |
| --- | --- | --- | --- | --- | --- | --- | --- | --- | --- | --- | --- | --- | --- | --- | --- |
| 1 | 1 | 1 | 1 | 1 | -1 | 1 | -1 | -1 | -1 | 1 | 1 | 1 | -1 | 1 | 1.16 |
| 2 | 2 | 1 | 1 | 1 | 1 | -1 | 1 | -1 | -1 | -1 | 1 | 1 | 1 | -1 | 3.88 |
| 3 | 3 | 1 | 1 | -1 | 1 | 1 | -1 | 1 | -1 | -1 | -1 | 1 | 1 | 1 | 2.7 |
| 4 | 4 | 1 | 1 | 1 | -1 | 1 | 1 | -1 | 1 | -1 | -1 | -1 | 1 | 1 | 2.02 |
| 5 | 5 | 1 | 1 | 1 | 1 | -1 | 1 | 1 | -1 | 1 | -1 | -1 | -1 | 1 | 3.82 |
| 6 | 6 | 1 | 1 | 1 | 1 | 1 | -1 | 1 | 1 | -1 | 1 | -1 | -1 | -1 | 2.76 |
| 7 | 7 | 1 | 1 | -1 | 1 | 1 | 1 | -1 | 1 | 1 | -1 | 1 | -1 | -1 | 3.8 |
| 8 | 8 | 1 | 1 | -1 | -1 | 1 | 1 | 1 | -1 | 1 | 1 | -1 | 1 | -1 | 2.52 |
| 9 | 9 | 1 | 1 | -1 | -1 | -1 | 1 | 1 | 1 | -1 | 1 | 1 | -1 | 1 | 2.12 |
| 10 | 10 | 1 | 1 | 1 | -1 | -1 | -1 | 1 | 1 | 1 | -1 | 1 | 1 | -1 | 0.96 |
| 11 | 11 | 1 | 1 | -1 | 1 | -1 | -1 | -1 | 1 | 1 | 1 | -1 | 1 | 1 | 2.76 |
| 12 | 12 | 1 | 1 | -1 | -1 | -1 | -1 | -1 | -1 | -1 | -1 | -1 | -1 | -1 | 0.86 |
| 13 | 13 | 1 | 1 | 1 | -1 | 1 | -1 | -1 | -1 | 1 | 1 | 1 | -1 | 1 | 1.14 |
| 14 | 14 | 1 | 1 | 1 | 1 | -1 | 1 | -1 | -1 | -1 | 1 | 1 | 1 | -1 | 3.86 |
| 15 | 15 | 1 | 1 | -1 | 1 | 1 | -1 | 1 | -1 | -1 | -1 | 1 | 1 | 1 | 2.68 |
| 16 | 16 | 1 | 1 | 1 | -1 | 1 | 1 | -1 | 1 | -1 | -1 | -1 | 1 | 1 | 1.94 |
| 17 | 17 | 1 | 1 | 1 | 1 | -1 | 1 | 1 | -1 | 1 | -1 | -1 | -1 | 1 | 3.88 |
| 18 | 18 | 1 | 1 | 1 | 1 | 1 | -1 | 1 | 1 | -1 | 1 | -1 | -1 | -1 | 2.76 |
| 19 | 19 | 1 | 1 | -1 | 1 | 1 | 1 | -1 | 1 | 1 | -1 | 1 | -1 | -1 | 3.76 |
| 20 | 20 | 1 | 1 | -1 | -1 | 1 | 1 | 1 | -1 | 1 | 1 | -1 | 1 | -1 | 2.46 |
| 21 | 21 | 1 | 1 | -1 | -1 | -1 | 1 | 1 | 1 | -1 | 1 | 1 | -1 | 1 | 2.06 |
| 22 | 22 | 1 | 1 | 1 | -1 | -1 | -1 | 1 | 1 | 1 | -1 | 1 | 1 | -1 | 0.92 |
| 23 | 23 | 1 | 1 | -1 | 1 | -1 | -1 | -1 | 1 | 1 | 1 | -1 | 1 | 1 | 2.74 |
| 24 | 24 | 1 | 1 | -1 | -1 | -1 | -1 | -1 | -1 | -1 | -1 | -1 | -1 | -1 | 0.94 |

| Variable | Code | Level (g/L) | | | | |
| --- | --- | --- | --- | --- | --- | --- |
|  |  | -1.414 | -1 | 0 | 1 | 1.414 |
| Fructose | A | 48.78 | 55 | 70 | 85 | 91.21 |
| Yeast extract | B | 12.1 | 15 | 25 | 29 | 31.89 |

Table S2. Specific levels for the CCD-RSM experiment in g/L. Code A is for fructose and B for yeast extract.

Table S3. Elemental analysis results of R2A media.

| Element | Average | Standard derivation | % Rel. S.D. | Variance |
| --- | --- | --- | --- | --- |
| Nitrogen | 4.5597 | 0.22 | 5.0078 | 0.0521 |
| Carbon | 36.3870 | 0.15 | 0.4159 | 0.0229 |
| Hydrogen | 5.8500 | 0.36 | 6.2941 | 0.1356 |
| Sulphur | 0.3644 | 0.03 | 8.3944 | 0.0009 |

Table S4. Experimental sets of RSM and responses and predicted values of biomass and lipids in g/L. The actual level indicates the concentration of the coded level in g/L.

|  | | Coded level | | | Actual level | | | Biomass response | | | Lipid response | |
| --- | --- | --- | --- | --- | --- | --- | --- | --- | --- | --- | --- | --- |
| Std. order | F | | Y | F | | Y | Actual | | Pred. | actual | | Pred. |
| 1 | -1 | | -1 | 55 | | 15 | 33.73 | | 35.26 | 5.18 | | 5.20 |
| 2 | 1 | | -1 | 85 | | 15 | 41.03 | | 41.93 | 5.89 | | 6.06 |
| 3 | -1 | | 1 | 55 | | 29 | 37.63 | | 38.85 | 5.82 | | 5.76 |
| 4 | 1 | | 1 | 85 | | 29 | 37.75 | | 38.34 | 5.40 | | 5.50 |
| 5 | -1.41 | | 0 | 48.79 | | 22 | 33.99 | | 32.38 | 5.15 | | 5.21 |
| 6 | 1.41 | | 0 | 91.21 | | 22 | 37.46 | | 36.74 | 5.77 | | 5.63 |
| 7 | 0 | | -1.41 | 70 | | 12.10 | 41.76 | | 42.63 | 5.87 | | 5.84 |
| 8 | 0 | | 1.41 | 70 | | 31.90 | 45.83 | | 42.63 | 5.90 | | 5.84 |
| 9 | 0 | | 0 | 70 | | 22 | 44.52 | | 46.01 | 6.27 | | 6.38 |
| 10 | 0 | | 0 | 70 | | 22 | 47.68 | | 46.01 | 6.27 | | 6.38 |
| 11 | 0 | | 0 | 70 | | 22 | 46.37 | | 46.01 | 6.62 | | 6.38 |
| 12 | 0 | | 0 | 70 | | 22 | 45.90 | | 46.01 | 6.58 | | 6.38 |
| 13 | 0 | | 0 | 70 | | 22 | 45.83 | | 46.01 | 6.16 | | 6.38 |

| Source | Sum of squares | Degree of freedom | Mean square | *F-Value* | *P-value* | Remarks |
| --- | --- | --- | --- | --- | --- | --- |
| Model | 2.44 | 5 | 0.49 | 14.86 | 0.0013 | Significant |
| A-Fructose | 0.17 | 1 | 0.17 | 5.17 | 0.0572 |  |
| B-Yeast extract | 0.00 | 1 | 0.00 | 0.13 | 0.7247 |  |
| AB | 0.31 | 1 | 0.31 | 9.55 | 0.0176 |  |
| A^2^ | 1.63 | 1 | 1.63 | 49.80 | 0.0002 |  |
| B^2^ | 0.53 | 1 | 0.53 | 16.03 | 0.0052 |  |
| Residual | 0.23 | 7 | 0.03 |  |  |  |
| Lack of fit | 0.06 | 3 | 0.02 | 0.51 | 0.6979 | Not significant |
| Pure error | 0.17 | 4 | 0.04 |  |  |  |
| Cor. total | 2.67 | 12 |  |  |  |  |
| R^2^ of reduced model is 0.9139, Adjusted R^2^ = 0.8524, Predicted R^2^ =0.7338 and adequate precision = 9.821 | | | | | | |

Table S5. ANOVA table for the lipid RSM model. The R-squared is 0.9139 and adequate precision is 9.139. The quadratic equation is y = 6.38 + 0.15 * F – 0.48 * F^2 – 0.27 * Y^2 – 0.28 * F * Y and the Optimal point is F = 72.7 g/L, YE = 21.3 g/L, with lipid concentration of 6.39 g/L.

Table S6. Productivity of heterotrophic cultivation including lipid content, lipid productivity, and biomass productivity of microalgae from heterotrophic cultivation.

| Species | Cultivation mode, carbon source | Lipid content (%) | Lipid Productivity (g/L/d) | Biomass productivity (g/L/d) | Working volume |  |
| --- | --- | --- | --- | --- | --- | --- |
| *Chlorella protothecoides* | Batch, sweet sorghum juice | 53 | 0.59 | 1.12 | 200 mL | Gao et al., 2010 ^33^ |
| *Chlorella protothecoides* | Batch, Jerusalem artichoke hydrolysate | 46 | 1.6 | 3.48 | 500 mL | Cheng et al., 2009 ^14^ |
| *Chlorella protothecoides* | Fed batch, sugarcane bagasse hydrolysate | 34 | 1.19 | 3.5 | 100 mL | Mu et al., 2015 ^34^ |
| *C. vulgaris* CCTCC M 209256 | Batch, enzyme hydrolysate of LEA | 35 | 0.12 | 0.33 | 1 L | Zheng et al., 2012 ^35^ |
| *Chlorella protothecoides* UTEX 25 | Fed batch, glycerol | 36 | 1.18 | 3.28 | 1.6 L | Ceron et al., 2013 ^32^ |
| *Chlorella protothecoides* UTEX 25 | Semi-continuous, glycerol | 50 | 4.3 | 8.6 | 1.6 L | Ceron et al., 2013 ^32^ |
| *Chlorella* sp. | Batch, molasses hydrolysate, LEA hydrolysate | 45 | 0.34 | 0.74 | 300 mL | Zheng et al., 2015 ^36^ |
| *Chlorella vulgaris* NIES-227 | Batch, glucose | 89 | 0.13 | 0.14 | 1 L | Shen et al., 2015 ^37^ |
| *Neochloris oleoabundans* UTEX 1185 | Exponential fed batch, glucose | 54 | 1.02 | 1.9 | 3 L | Morales et al., 2014 ^11^ |
| *Ettlia* sp. YC001 | Batch fermenter, fructose | 16.4 | 1.18 | 7.21 | 3 L | This work |

Figure S1. Design points of validation experiment.


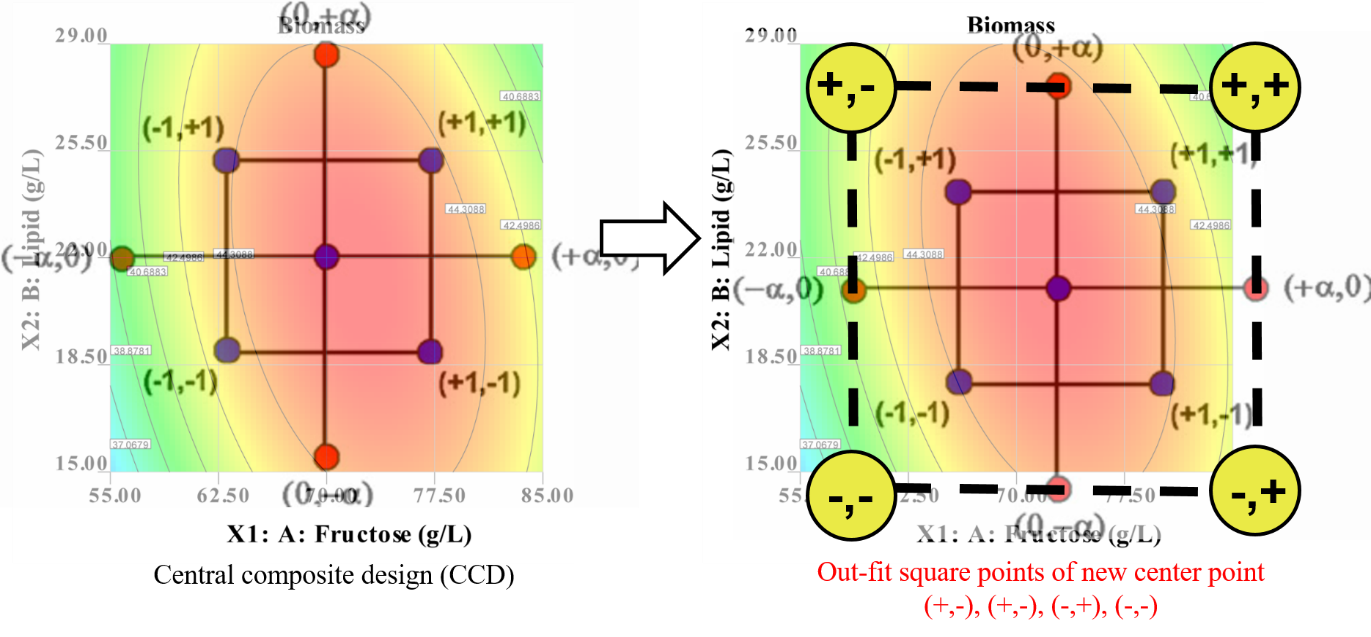


Figure S2. RSM result in a 3D surface (a) and a contour (b) for the lipid response. The interaction of fructose concentration and yeast extract concentration is represented in g/L. The peak of the surface is the optimal point, which is located at fructose 72.4 g/L, yeast extract 21.7 g/L, with a lipid response of 6.4 g/L (desirability 0.844).

**(b)**

**(a)**


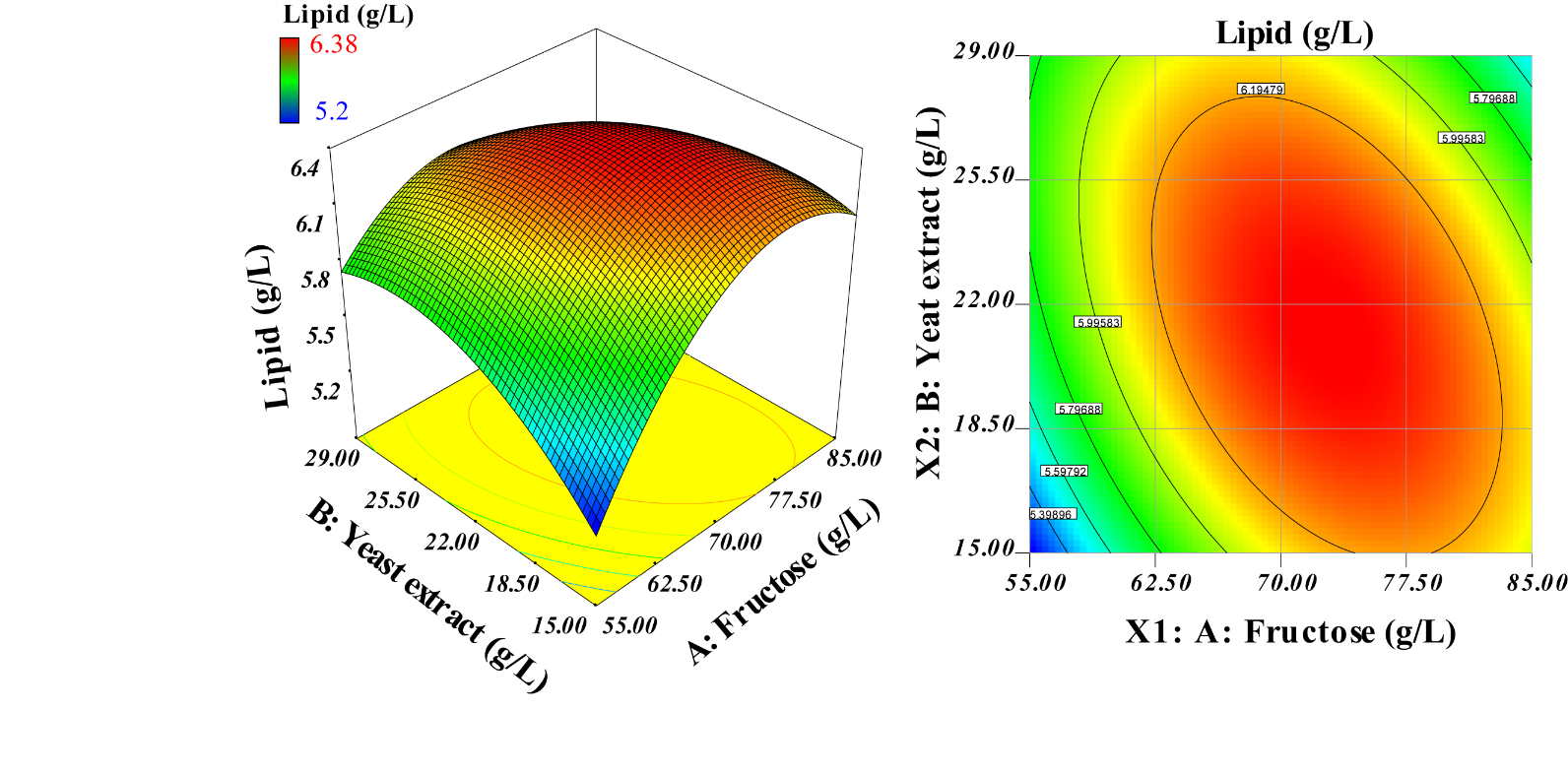

Supplement: Supplementary file 1 — Supplementary information [file 41598_2019_43366_MOESM1_ESM.docx]
